# Validity and reliability of the 2-minute walk test in individuals with spinal cord injury

**DOI:** 10.1038/s41393-022-00847-1

**Published:** 2022-08-23

**Authors:** Romina Willi, Mario Widmer, Nora Merz, Caroline H. G. Bastiaenen, Björn Zörner, Marc Bolliger

**Affiliations:** 1 Spinal Cord Injury Centre, Balgrist University Hospital Zurich, University of Zurich, Zurich, Switzerland; 2 Department of Therapy, Swiss Paraplegic Centre, Nottwil, Switzerland; 3 Department of Epidemiology, Maastricht University, Maastricht, The Netherlands

**Keywords:** Spinal cord diseases, Spinal cord diseases

## Abstract

**Study design:**

Multicentre-observational study.

**Objectives:**

The 6-minute walk test (6mWT) is an established assessment of walking function in individuals with spinal cord injury (SCI). However, walking 6 min can be demanding for severely impaired individuals. The 2-minute walk test (2mWT) could be an appropriate alternative that has already been validated in other neurological disorders. The aim of this study was to assess construct validity and test-rest reliability of the 2mWT in individuals with SCI. In addition, the influence of walking performance on sensitivity to change of the 2mWT was assessed.

**Setting:**

Swiss Paraplegic Center Nottwil, Switzerland; Balgrist University Hospital, Zürich, Switzerland.

**Methods:**

Fifty individuals (aged 18–79) with SCI (neurological level of injury: C1-L3, AIS: A-D) were assessed on two test days separated by 1 to 7 days. The first assessment consisted of a 2mWT familiarization, followed by a 2mWT and 10-meter walk test (10MWT) (including the Walking Index for Spinal Cord Injury (WISCI II)) in randomized order. The second assessment consisted of 2mWT and 6mWT in randomized order. Tests were separated by at least 30 min of rest.

**Results:**

The interclass correlation coefficient between the 2mWT assessed on the first and second test day was excellent (*r* = 0.980, *p* < 0.001). The 2mWT correlated very strongly with the 6mWT (*r* = 0.992, *p* < 0.001) and the 10MWT (*r* = 0.964, *p* < 0.001), and moderately with the WISCI II (*r* = 0.571, *p* < 0.001). Sensitivity to change was slightly affected by walking performance.

**Conclusion:**

The 2mWT is a valid and reliable alternative to the 6mWT to measure walking function in individuals with SCI.

**Trial registration:**

NCT04555759.

## Introduction

Almost half of all spinal cord injuries (SCI) are functionally incomplete [1, 2], meaning that there is some remaining motor or sensory function below the level of the lesion [3]. These individuals have a high chance to regain some walking function after rehabilitation [1, 4, 5]. But also a small proportion of individuals with complete thoracolumbar SCI are able to recover some lower extremity function [6]. Regaining locomotor function after SCI is of high priority for the affected individuals, especially in individuals with paraplegia [7]. This shows the need for valid and reliable gait assessments that allow therapists to monitor individuals’ walking performance during the course of rehabilitation [8].

One of the most established assessments of walking function in individuals with SCI is the 6-minute walk test (6mWT) which measures the distance an individual is able to walk within 6 min. The 6mWT is widely used to measure endurance, fatigability, and cardiovascular fitness [9], showing good psychometric properties in individuals with SCI [8, 10–12]. However, performing the 6mWT is time-consuming, a critical factor in daily clinical practice. Moreover, walking 6 min can be demanding for severely impaired individuals resulting in a floor effect [13]. To overcome these limitations, the 2-minute walk test (2mWT) has gained more attention in the SCI field. Derived from the 6mWT, the 2mWT assesses walking performance by measuring the distance a person is able to walk within 2 min. Previous studies have shown a strong association between the 6mWT and the 2mWT in other neurological disorders like multiple sclerosis [14], stroke [15], and neuromuscular disease [16], but psychometric properties of the 2mWT have not yet been established for the SCI population. Therefore, this study aimed to investigate the construct validity of the 2mWT as an outcome measure for walking function in individuals with SCI by testing its relationship with other commonly used functional tests. In addition, test-retest reliability of the 2mWT was assessed by quantifying the degree to which two measurements performed within 1 week are free from measurement error [17], which means in other words, to quantify the measurement error.

Variability in gait performance is expected to be greater in individuals with a lower walking function [16]; for example, in stroke survivors, SEM and MDC are influenced by walking speed [18]. Therefore, we were also interested in whether the construct validity and the test-retest reliability, as well as standard error of measurement (SEM) and minimal detectable change (MDC) are affected by gait performance in individuals suffering from SCI.

We hypothesize that (1) the 2mWT will show a very strong correlation of 0.8–0.99 with the 6mWT, (2) the 2mWT will show a good to excellent test-retest reliability, ICC higher than 0.8, (3) the SEM and the MDC are influenced by the walking function, (4) the walking function has no influence on the correlation between the 2mWT and the 6mWT (slow and fast walkers: *r* = 0.8–0.99), and (5) the distance walked per minute will decrease over time in the 6mWT but not the 2mWT.

## Methods

### Study population

For this multicentre study, individuals with SCI were recruited at the Spinal Cord Injury Center Balgrist and the Swiss Paraplegic Center Nottwil. The inclusion criteria were: acute or chronic SCI, ≥18 years and a minimum walking speed of 0.17 m/s (~corresponding to the ability to perform a 10-meter walk test (10MWT) in <60 s), and ability to walk without physical assistance. We excluded individuals who walked <60 meters in 6 min due to a potential high variability of walking performance as this may occur in individuals with poor walking function [16]. The exclusion criteria were: current orthopedic problems, major psychosis or depression, and history of severe heart condition.

### Procedure

This study was approved by the Ethical Committee of the Canton of Zurich and the Ethics Committee for Northwest/Central Switzerland (BASEC 2020-01473) and was conducted in accordance with Good Clinical Practice guidelines and the Declaration of Helsinki. Prior to enrollment, written informed consent was obtained from all participants. Individuals were invited to participate on two test days, separated by 1–7 days. A maximum of 7 days has been chosen because it can be assumed that the walking function remains stable during this time period. On the first day, a familiarization run of the 2mWT was performed. After a break of at least 30 min, the 2mWT and the 10MWT were performed in randomized order, again separated by at least 30 min of rest. The WISCI II was applied and scored based on the performance in the 10MWT. The second testing day consisted of 2mWT and 6mWT, performed in randomized order and separated by a 30 min break.

### Measures

Examiners from both centers were trained all together in performing the assessments to optimize the standardized collection of all outcome measures.

#### 2mWT and 6mWT

The walk tests were performed according to the Guidelines of the American Thoracic Society [19], except that the hallway length was 35 meters (instead of 30 meters) in both study centers. An examiner accompanied each subject for safety reasons, walking behind the subject to allow them to set the pace.

Any braces and/or habitual assistive devices were permitted but needed to be kept consistent on both testing days. Subjects were instructed to walk as far as possible, but safely, during the respective test time of 2 or 6 min. Standardized instructions were read out to the participants. Every minute during the test, they were informed about the remaining time and were encouraged to keep up the good work. No other communication occurred during the test.

Subjects were allowed to take rest breaks if needed, but time continued to run during the break.

The 6mWT has shown very good construct validity (10MWT: *r* = 0.86) [8] and excellent test-retest reliability (ICC = 0.99) [12] in individuals with chronic SCI.

#### 10MWT

The 10MWT was performed with a flying start, i.e., subjects were instructed to walk a total distance of 14 meters including two meters to accelerate and two meters to decelerate. Time was measured for the middle ten meters and rounded to the next tenth of a second.

Participants performed a total of four 10MWT runs: two at their preferred walking speed (10MWT self), and two at their maximal walking speed (10MWT max), i.e., as fast as possible, but still safe. The average of the two runs each was taken as the final result for each variant.

The 10MWT has shown very good construct validity (TUG: *r* = 0.89; 6mWT: *r* = 0.95) [20] and excellent test-retest reliability (ICC = 0.983 [20]) in individuals with SCI.

#### WISCI II

The WISCI II is an ordinal scale to assess walking capacity [21]. It captures the extent and nature of assistance a person with SCI requires to walk ten meters. This assessment index includes a rank ordering along a dimension of impairment, from the level of most severe impairment (level 0) to least severe impairment (level 20). The level is based on the use of devices, braces, and physical assistance of one or more persons. Each successive level is a less impaired level than the former. The ranking is based on the severity of the impairment and not on functional independence in the environment.

For the present study, the WISCI II was scored based on the performance of the 10MWT.

The WISCI II has shown good construct validity (TUG: 0.76; 10MWT: 0.68; 6mWT: 0.60) [8] and excellent test-retest reliability (self-selected WISCI: ICC = 0.994; maximal WISCI: ICC = 0.995) [22].

### Statistics

The sample size of 50 was chosen based on the COSMIN guidelines [17].

There was no missing data as, in accordance with the study protocol, the three drop-outs have been replaced. No further cleaning was necessary since all datasets were complete and plausible. Data were analyzed using RStudio for Mac, version 1.3.1093. Descriptive data are reported using mean ± standard deviation or median (range).

#### Construct validity

Data were tested for normality using the Shapiro–Wilk test. If data were normally distributed, construct validity was tested using the Pearson correlation. Otherwise, a Spearman rank correlation was applied.

According to the guide from Evans et al. [23], the following ranges for the correlation coefficient (*R*) were used for the interpretation of the strength of the association: 0.00–0.19: “very weak”, 0.20–0.39: “weak”, 0.40–0.59: “moderate”, 0.60–0.79: “strong” and 0.80–1.0: “very strong”.

Based on studies in other neurological populations [14–16], a “very strong” Pearson correlation between the 2mWT and the 6mWT was predefined as requirement to recommend the use of the 2mWT for routine clinical use in individuals with SCI.

#### Test-retest reliability

Test-retest reliability was determined by calculating the interclass correlation coefficient (ICC) (two-way mixed effects, total agreement) between the 2mWT from the first and the second test day [24]. To interpret the ICC values, we have referred to the recommendation from Koo et al. [24], <0.5: “poor”, 0.5–0.76: “moderate”, 0.75–0.9 “good” and >0.9 “excellent”. ICCs > 0.8 are acceptable for clinical work [25].

In addition, a Bland–Altman plot was created to estimate the absolute agreement of the two measurements of the 2mWT.

The SEM $\left( {{{{{{\rm{SEM}}}}}} = {{{{{\rm{SD}}}}}} \times \sqrt {\left( {1 - {{{{{\rm{ICC}}}}}}} \right)} } \right)$ [26] and the MDC $\left( {{{{{{\rm{MDC}}}}}} = {{{{{\rm{SEM}}}}}} \times 1.96 \times \sqrt 2 } \right)$ [27] were calculated based on the respective formulas.

#### Minute intervals

A paired *t*-test was used to compare the distances walked in the 2mWT in the first and the 2nd min. Moreover, a Friedman test was applied to compare the minute intervals of the 6mWT with paired post-hoc Wilcoxon Tests (Bonferroni corrected), if applicable. A significance level of *p* < 0.05 was selected.

#### Subgroup analysis

Two subgroup analysis were performed: (1) the influence of time since injury on construct validity and test-retest reliability and (2) the influence of walking performance on SEM and MDC was analyzed, each by creating two subgroups.

1. Although the maximal time window for the two test days was narrow with 7 days it cannot be excluded that individuals with a sub-acute SCI could show a spontaneous improvement in walking performance within this time window. Therefore, the study sample was divided into an acute/sub-acute (0–6 months after injury) and a chronic (>6 months after injury) SCI group.
2. To investigate whether SEM and MDC are influenced by the walking performance, the study sample was separated into slow and fast walkers. Community ambulation is often measured at crosswalks, where speed is the primary concern [28]. In the US the recommended walking speed to safely cross an intersection range from 0.9 to 1.2 m/s [29]. Since the median of the speed in the 2mWT of our sample was 0.9 m/s we decided to use this as cut off to divide our sample into two groups.

## Results

### Participants characteristics

Fifty-three individuals with SCI were recruited for this study. Three individuals had to be excluded, two due to medical reasons (after screening) and one was not able to complete the 6mWT due to pain during walking. As specified in the protocol, these drop-outs have been replaced, resulting in the a priori targeted sample of 50. Descriptive details about the participant characteristics can be found in Table 1.

**Table 1:** Descriptive characteristics and performance on the gait measures.

Variable	Value
Age, years	52.6 ± 16.2
Gender	
Male	33 (66)
Female	17 (34)
Height, cm	174.7 ± 10.1
Weight, kg	76.6 ± 16.7
NLI	
Tetraplegic	24 (48)
Paraplegic	26 (52)
AIS Grade	
A	2 (4)
B	0 (0)
C	7 (14)
D	41 (82)
Type of injury	
Traumatic	28 (56)
Non-traumatic	22 (44)
Year since injury	6.11 ± 9.8
2-minute walk test familiarization [m]	107.9 ± 52.6
2-minute walk test day 1 [m]	114.1 ± 53.3
2-minute walk test day 2 [m]	118.8 ± 54.0
6-minute walk test [m]	338.2 ± 165.8
Self 10-meter walk test [s]	15.2 ± 10.0
Max 10-meter walk test [s]	11.4 ± 7.6
WISCI II (range)	8–20

Data are presented as mean ± SD or number (percentage).

*NIL* neurological level of injury, *AIS* American Spinal Injury Association Impairment Scale, *WISCI II* Walking Index for Spinal Cord Injury II.

### Construct validity

**Fig. 1:**
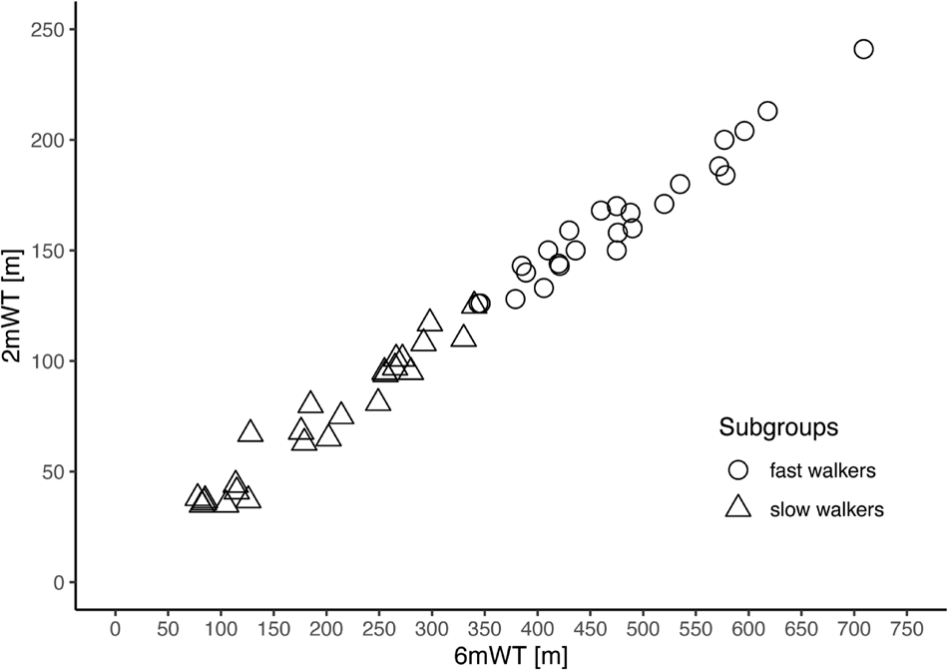
Correlation between 2-minute walk (2mWT) test and 6-minute walk test (6mWT). Distances walked in 2mWT and 6mWT. Individuals grouped into fast walkers (circles) and slow walkers (triangles).

The 2mWT showed very strong correlations with the 6mWT (Fig. 1 and Table 2) and the 10MWT and a moderate relationship with the ordinal score of the WISCI II (Table 2). The dimension of the correlations for the 2mWT and the 6mWT with the other assessments were comparable.

**Table 2:** Walk test distances and other functional measures.

		6mWT	Self 10MWT	Max 10MWT	WISCI II
Overall	2mWT	0.992 (0.986–0.995)	0.964 (0.941–0.986)	0.974 (0.956–0.988)	0.571 (0.356–0.784)
	6mWT	–	0.959 (0.928–0.989)	0.985 (0.975–0.993)	0.580 (0.368–0.792)
	Self 10MWT		–	0.958 (0.925–0.989)	0.587 (0.372–0.799)
	Max 10MWT			–	0.538 (0.301–0.764)
Slow	2mWT	0.973 (0.938–0.988)	0.932 (0.884–0.973)	0.925 (0.856–0.9881)	0.093 (−0.415–0.600)
Fast	2mWT	0.974 (0.941–0.987)	0.845 (0.702–0.981)	0.893 (0.7815–0.996)	0.427 (0.0623–0.787)

Values in parentheses are 95% CIs.

*2mWT* 2-minute walk test, *6mWT* 6-minute walk test, *10MWT* 10-meter walk test, *WISCI II* Walking Index for Spinal Cord Injury II.

### Test-retest reliability

**Fig. 2:**
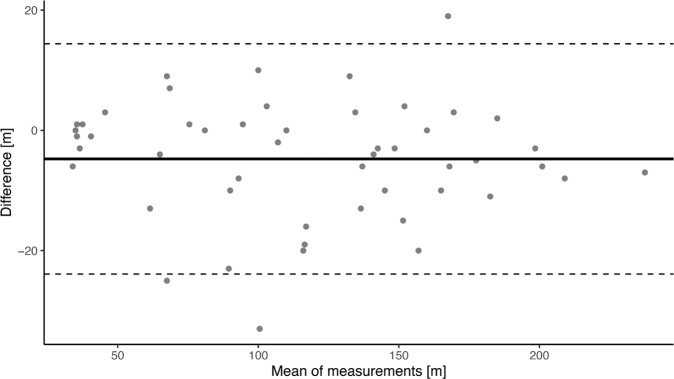
Bland Altman plot to assess test retest reliability. The averages of the two measurements were plotted against the differences. The closer the points are located to the horizontal solid line the better the reliability. The dashed lines represent the 95% limits of agreement.

The walking distance of the 2mWT on the first and second test day showed a very high ICC (ICC = 0.980, *p* < 0.001). The Bland–Altman plot (Fig. 2) is illustrating the test-retest-reliability by plotting the average against the differences of the two measurements. The plot revealed a systematic error of 4.74 meters. A comparison of means showed that the walked distance on the second day is significantly different compared to the walked distance on the first day (*p* < 0.001). The SEM and the MDC are presented in Table 4.

### Walked distance

The mean distance walked for the 2mWT was 114.1 ± 53.3 meters on the first day and 118.8 ± 54.0 meters on the second day. For the 6mWT, the average walking distance was 338.2 ± 165.8 meters. Walking distances during 1-min intervals of the 2mWT were not different (Day 1: *t* = 1.4406, df = 49, *p* = 0.1561; Day 2: *t* = 0.74822, df = 49, *p* = 0.4579).

**Fig. 3:**
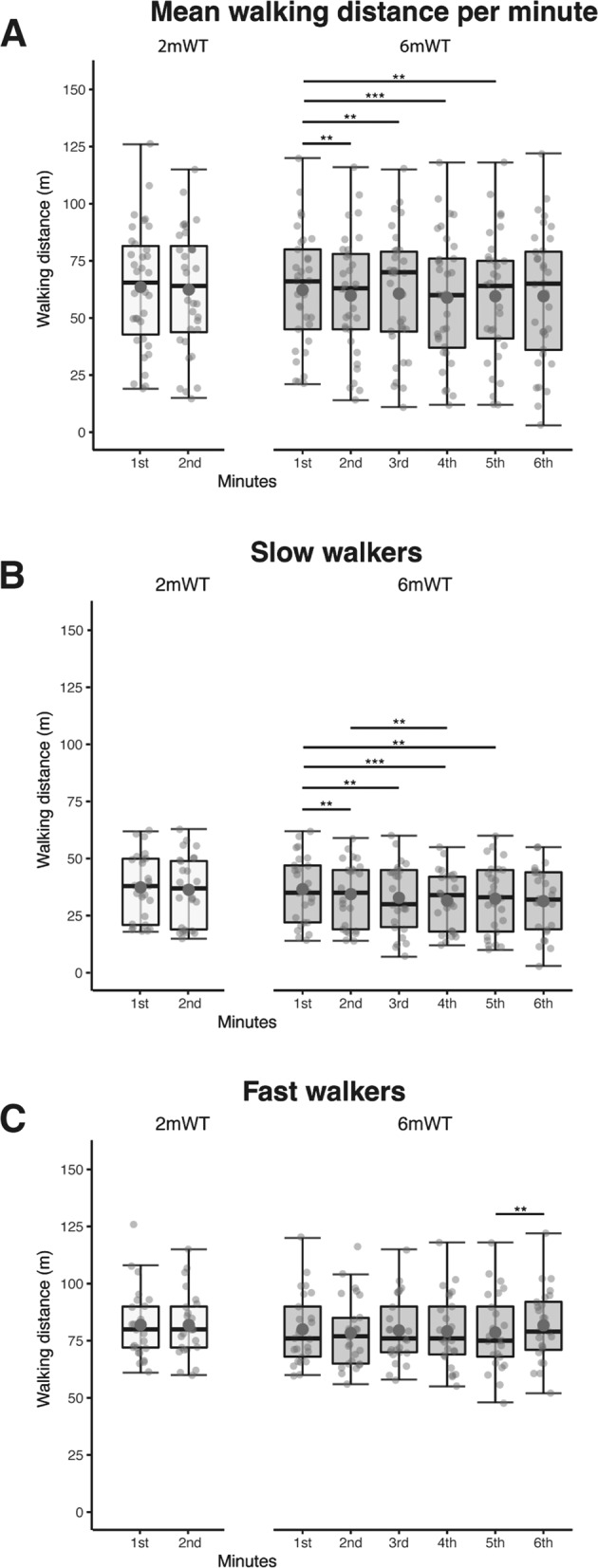
Minute intervals of 2mWT and 6mWT. Walked distances during minute intervals from the 2-minute walk test (2mWT) and the 6-minute walk test (6mWT) overall (**A**) and when separated in slow (**B**) and fast walkers (**C**).

In the 6mWT, minute intervals differed significantly (Friedman chi-squared = 41.285, df = 5, *p* < 0.001), with all intervals being significantly different from the first minute except for the sixth minute (Fig. 3). Projecting the distance walked during 6 min by multiplying the result from the 2mWT by three (3 × 2mWT = 356.5 m) overestimated the actual distance of the 6mWT (*t* = −6.1115, df = 49, *p* < 0.001, mean difference −18.24 m), since the velocity in the 6mWT is lower than in the 2mWT.

### Subgroup comparisons

Construct validity and test-retest reliability were not affected by time since injury (Supplementary Table S1). Hence, time since injury was not further considered for the interpretation of the results.

The fast walkers walked on average more than twice as far as the slow walkers in the 2mWT (slow walkers: 73.8 m; fast walkers: 163.8 m) and the 6mWT (slow walkers: 199.0 m; fast walkers: 477.4 m). The correlation between the 2mWT and the 6mWT remained, even when splitting into subgroups (*r* = 0.973 for the slow walkers and *r* = 0.974 for the fast walkers, both *p* < 0.001). Correlation coefficients of the 2mWT with the other assessments can be found in Table 2.

In addition, test-retest reliability was preserved for each subgroup (ICC = 0.923 and 0.941 for the slow and fast walkers (both *p* < 0.001)). Results of the different gait tests divided into the two subgroups are presented in Table 3.

**Table 3:** Performance on gait measures.

Variable	Slow walkers	Fast walkers
2-minute walk test familiarization [m]^a^	64.4 ± 27.3	151.5 ± 30.9
2-minute walk test day 1 [m]^a^	69.5 ± 27.2	158.7 ± 30.3
2-minute walk test day 2 [m]^a^	73.8 ± 29.6	163.8 ± 29.0
6-minute walk test [m]^a^	199.0 ± 85.2	477.4 ± 92.1
Self 10-meter walk test [s]^a^	21.8 ± 10.4	8.5 ± 1.5
Max 10-meter walk test [s]^a^	16.4 ± 8.0	6.5 ± 1.3
WISCI II (range)^a^	14.2 ± 3.2 (8–20)	17.8 ± 3.2 (12–20)

Data are presented as mean ± SD.

*WISCI II* Walking Index for Spinal Cord Injury II.

^a^Significant difference between the groups at *p* < 0.001.

There was no significant difference in the distances walked per minute in the 2mWT in any of the subgroups. However, there were significant differences in the 6mWT (Slow walkers: Friedman chi-squared = 41.285, df = 5, *p* ≤ 0.001; fast walkers: Friedman chi-squared = 19.285, df = 5, <0.01) (Fig. 3B, C).

The calculation of SEM and MDC for the subgroups showed that these values are influenced by gait performance. The values for the slow walkers are slightly higher than for the fast walkers (see Table 4).

**Table 4:** Standard error of measurement and minimal detectable change for the 2mWT.

	SEM	MDC
Overall	7.5 m	20.9 m
Slow walkers	8.5 m	23.6 m
Fast walkers	7.3 m	20.3 m

*SEM* standard error of measurement, *MDC* minimal detectable change.

## Discussion

About one-third of all individuals with SCI are able to walk (at least) one block (~80 meters) 1 year post injury [30]. Hence, there is a need for valid and reliable assessments to measure and monitor walking performance in individuals with SCI. These can be used to evaluate the efficacy of rehabilitation interventions targeting walking function in this population. This study aimed at assessing the construct validity and test-retest reliability of the 2mWT in individuals with SCI.

The main findings of the present study are that in individuals with SCI (1) the walking distance assessed in the 2mWT and the 6mWT are highly correlated, (2) the 2mWT has an excellent test-retest reliability, (3) SEM and MDC are different for fast and slow walkers, (4) the walking function has only a minor effect on the correlation between the 2mWT and the 6mWT, and (5) the 2mWT cannot capture exhaustion.

It has been shown that the 2mWT correlates strongly with the 6mWT in individuals with neuromuscular disease [16], multiple sclerosis [14], and stroke [15]. This is consistent with our results in individuals with sub-acute and chronic SCI. Based on the strong correlation between the 2mWT and the 6mWT, Gijbles et al. [14] speculated that both tests capture the same aspects of mobility. However, in individuals with neuromuscular disease, the 6mWT is affected by motor fatigue (reflected in a decrease of walking speed toward the end of the testing time) while the 2mWT is too short to capture motor fatigue [16]. We observed a similar pattern in individuals with SCI. The 2mWT was too short to capture motor fatigue in both, fast and slow walking individuals with SCI, but we observed a significant decrease in walking speed over the course of the 6mWT in the slow walker subgroup. However, these variations in walking speed could also be attributed to motivational aspects, or a strategic approach to the assessment, which might be more pronounced for longer walking periods [16]. Whether the 6mWT provides more information about the fatigability of the subjects therefore remains unclear [16]. In contrast to the slow walkers, the fast walkers even accelerated for the last-minute balancing out the decreased walking speed of the slow walkers and hence, resulting in no significant difference between the first and the last minute of the 6mWT when both groups are taken together in the overall sample.

It should be noted that generally only final distances (and not minute intervals) are documented for these tests, e.g., during clinical routine, but also most of the research reports. If this is the case, the 2mWT is a valid alternative to the 6mWT to describe walking function in individuals with SCI, given the very high correlation coefficients for the overall patient sample, but also for the slow and fast walker subgroups separately.

The subgroup analysis further revealed that the test-retest reliability of the 2mWT is slightly lower in the slow walkers since they tend to show more day to day variability in their walking performance. The slightly lower ICC of the slow walkers is also reflected in a slightly increased SEM and MDC. This underlines that it is important to calculate SEM and MDC separately for slow and fast walkers in functional tests.

In this study, we investigated test-retest reliability by comparing the distance of 2mWTs that were performed 1–7 days apart. Previous studies have shown that there is a learning effect in repeated walking tests [31]. To prevent this learning effect, our participants performed a familiarization trial before the very first assessment of the first testing day. Still, the participants covered a marginal larger distance in the 2mWT on the second day. The finding of increased walking distances in repeated walking tests is well known and has been described in other studies [32, 33]. However, the difference of 4.74 meters is smaller than the MDC of the 2mWT.

We excluded individuals with a walking distance <60 meters in 6 min. This is a limitation of the study since our results may not apply to individuals with more severely impaired walking function.

Moreover, we used a hallway length of 35 meters instead of the 30 meters suggested in the Guidelines of the American Thoracic Society [34]. However, a recent multicentre study found no significant effect of the length of straight courses ranging from 50 to 164 feet (15 –50 m) [35].

In the present study we did not consider all aspects of responsiveness (e.g., internal responsiveness) but the proper assessment of responsiveness would be critical in order to use the 2mWT to measure the potential impact of interventions on walking function over time in individuals with SCI. We can see from our data that the 2mWT will be more responsive in detecting walking function improvements in fast walkers, as they show less day to day variability and therefore smaller SEM and MDC than in slow walkers. Further research is required to determine all aspects of responsiveness of the 2mWT in measuring change over time or effects of interventions in individuals with SCI.

## Conclusion

Reliable, valid, and practicable assessments of walking function that are easy to use are of great importance, in the clinic and in research. This study demonstrated good to very good construct validity and excellent test-retest reliability of the 2mWT in individuals with SCI. Based on these findings, the 2mWT can be suggested as a suitable alternative to the 6mWT in individuals with SCI comparable to the investigated group. Its short application time may allow clinicians and researchers to measure walking function efficiently.

## Supplementary information

Supplementary Table S1

## Data Availability

The datasets generated and/or analyzed during the current study are available from the corresponding author on reasonable request.
