# Supplementary material for: Validity and reliability of the 2-minute walk test in individuals with spinal cord injury
Source: Spinal Cord. 2022 Aug 23;61(1):15–21. doi: 10.1038/s41393-022-00847-1 (PMC9836932; doi:10.1038/s41393-022-00847-1)
Supplement: Supplementary file 1 — Supplementary Table S1 [file 41393_2022_847_MOESM1_ESM.pdf]

Supplementary table S1: Construct validity and test-retest reliability between individuals with an acute and a chronic SCI

	n	Days after injury (range)	Days between measurements (mean ± stdev)	ICC	Corr. 2mWT and 6mWT	Corr. speed 2mWT and speed self-selected 10MWT	Corr. 2mWT and WISCI II
Acute/sub-acute	21	(19 - 183)	4.3 ± 2.0	0.982	0.991 (0.978 to 0.996)	0.933 (0.797 to 0.975)	0.465 (0.797 to 0.975)
Chronic	29	(219 - 16050)	4.1 ± 2.1	0.979	0.993 (0.985 to 0.997)	0.979 (0.797 to 0.975)	0.628 (0.262 to 0.830)

Note: Values in parentheses are 95% CIs.
Abbreviations: 2mWT, 2-Minute Walk Test; 6mWT, 6-Minute Walk Test; 10MWT, 10-Meter Walk Test; WISCI II, Walking Index for Spinal Cord Injury II
Acute/sub-acute: 0-6 months; chronic: > 6 months.
